# The Role of Temporal Abundance Structure and Habitat Preferences in the Survival of Conodonts during the Mid-Early Silurian Ireviken Mass Extinction Event

**DOI:** 10.1371/journal.pone.0124146

**Published:** 2015-04-10

**Authors:** Andrej Spiridonov, Antanas Brazauskas, Sigitas Radzevičius

**Affiliations:** Department of Geology and Mineralogy, Vilnius University, M. K. Čiurlionio 21/27, LT-03101 Vilnius, Lithuania; Universität Göttingen, GERMANY

## Abstract

The Ireviken event was one of the most intense extinction episodes that occurred during the mid-Paleozoic era. It had a strong global effect on a range of clades, with conodonts, graptolites and chitinozoans affected most. Using geophysical proxies and conodont species parameters of their temporal abundance structure we investigate how they affected the selectivity of conodont species survival during this calamity. After performing bivariate logistic analyses on 34 species of conodonts, we find three variables that were statistically significantly associated with their odds of survival. These namely include spectral exponents that describe degrees of autocorrelation in a time series, the skewness of species abundance distribution, and average environmental preferences, which are mostly determined by ancient water depths at sampling sites. Model selection of multivariate logistic models found the best model includes species local abundance skewness and substrate preference. A similar pattern is revealed through the regression tree analysis. The apparent extinction selectivity points to a possible causes of environmental deterioration during the Ireviken event. The significant positive relationship between extinction risk and preferential existence in deeper environments suggests the open ocean causal mechanisms of biotic stress that occurred during the Ireviken event. Marine regressions, which were previously suggested as a causal factor in this extinction episode, on theoretical grounds should have had higher impact on species living in near-shore environments, through the processes of habitat loss which are associated with decreases of shelfal areas. In addition, the significant positive correlations found between skewness of abundance distributions and spectral exponent values and the probability of species survival suggest that community and ecosystem processes (which controlled species abundance fluctuation patterns) were significantly related to selectivity processes of this smaller mass extinction event.

## Introduction

This study focuses on the Ireviken event, which is also known as the mid-early Silurian event. This event was one of the most profound macroevolutionary and environmental turnover events of the mid-Paleozoic period. As a critical geobiological episode, it was first recognized in the early 1990s by Lennart Jeppsson, who examined fossil records of early Silurian conodonts in Gotland (Sweden) Island [1, 2, 3, 4]. Based on global compilations (though the continuous development of taxonomy for this group does not allow for very high estimation precision), he found that 20% (12 out of 60) of conodont species passed continuously through the Ireviken while other species went extinct [4]. Highly detailed (cm scale) stratigraphy records show that this extinction event progressed in a step-like pattern through eight “datum” points (or turnover events). Each turnover event signifies a conodont species extinction or temporal extirpation event. Since this study was published, this event has been thoroughly researched by conodont workers based in other localities of the Baltica and in other paleocontinents and terrain regions [3,5,6,7]. These additional studies also supported the step-like pattern with two to three more turnover events [6]. The effects of the Irevikent event were not restricted to conodonts, however. Rather, significant extinction events occurred among other groups of marine biota to [8]. Severe losses were suffered in the graptolite clade, with extinction rates approaching 64% [9]. Though more recent estimates show relatively smaller drop of graptolite diversity by 50% [10]. Trilobites were also strongly affected, with extinction rates approaching 50%, according to Swedish material [4]. High resolution, quantitative biostratigraphic studies of chitinozoans from the eastern section of the Baltic basin deem the Ireviken event the most devastating extinction episode for this clade during the entire Silurian period, causing a 50% decline in standing diversity [11]. Polychaetes (scolecodonts) suffered a 20% loss of generic diversity in the Baltic due to extinction and temporal regional extirpation [12]. While knowledge on the full impact of the Ireviken event on polychaete fauna is still incipient, the severe influence of this event on these communities structure is evident [13]. Microplankton, according to material from Gotland, were affected to a lesser extent, with 18% of species (12 out of 72) eliminated due to extinction and extirpation [14] and similar patterns have been observed for the brachiopods [15]. Ireviken perturbation in the former group more closely resembled a turnover pulse (sensu [16]) macroevolutionary event than a true mass extinction event, as the event produced a net increase in palynomorph diversity [14].

From the distribution of extinction magnitudes, it is evident that taxonomically and ecologically differing groups were selectively affected. This finding is supported by observations of abundance change in key Estonian regions, with conodonts shown to be affected most and at the earliest periods [17]. It was also found that profound, global environment-altering geobiological effects of the Ireviken event postponed changes in the stable carbon isotopic (δ^13^C) composition of sedimentary rocks, generating a rise in mean values among Gotland outcrops from 1.4 ‰ to 4.5 ‰ [18] and maximal values of excursion reaching 6.6 ‰, as detected in Storøya (Oslo region) [19].

Environmental and biotic turnover patterns during the mid-early Silurian event have been documented in an array of research studies. Some studies based on analyses of marine organism genera have argued that the Silurian as a whole was characterized by low levels of evolutionary turnover and extinction impact on taxa [20]. However, extinction severity is not necessarily proportional across hierarchical levels, and this proportionality and its sign depends on extinction selectivity patterns or a lack thereof [21]. For example, given the widely recognized empirical observation that most genera are “species poor” [22, 23], if extinction episodes preferentially eliminate few “species-rich” genera, according to generic counts, species extinctions will be strongly underestimated. On the other hand, if “species-poor genera” are targeted more intensely, “species level” extinction would appear more severe than “genus level extinction”. All intermediates are also possible.

Despite considerable available information on the effect of the Ireviken event on life history, research remains underdeveloped. The phylogenetically, multi-cladal and geographically global impacts of the Ireviken event are evident [24]. This suggests that the event constituted an episode of true, though less extensive, mass extinction event.

The determinants of survival during the Ireviken event are poorly understood because the number of surviving conodont species is quite low [4]. Hence, rigorous statistical analyses are necessary in this area. Fitness differences usually play a fairly limited role during major mass extinction events, during which stochastic survival is of greater importance [21,25]. However, factors that facilitated species survival may have had distinct importance during turnover regimes of varying intensities. Accordingly, the Ireviken event, due to its smaller overall magnitude of impact on biota, may have had distinct macroevolutionary effects in comparison to the more conventional and devastating “big five” events.

**Fig 1:**
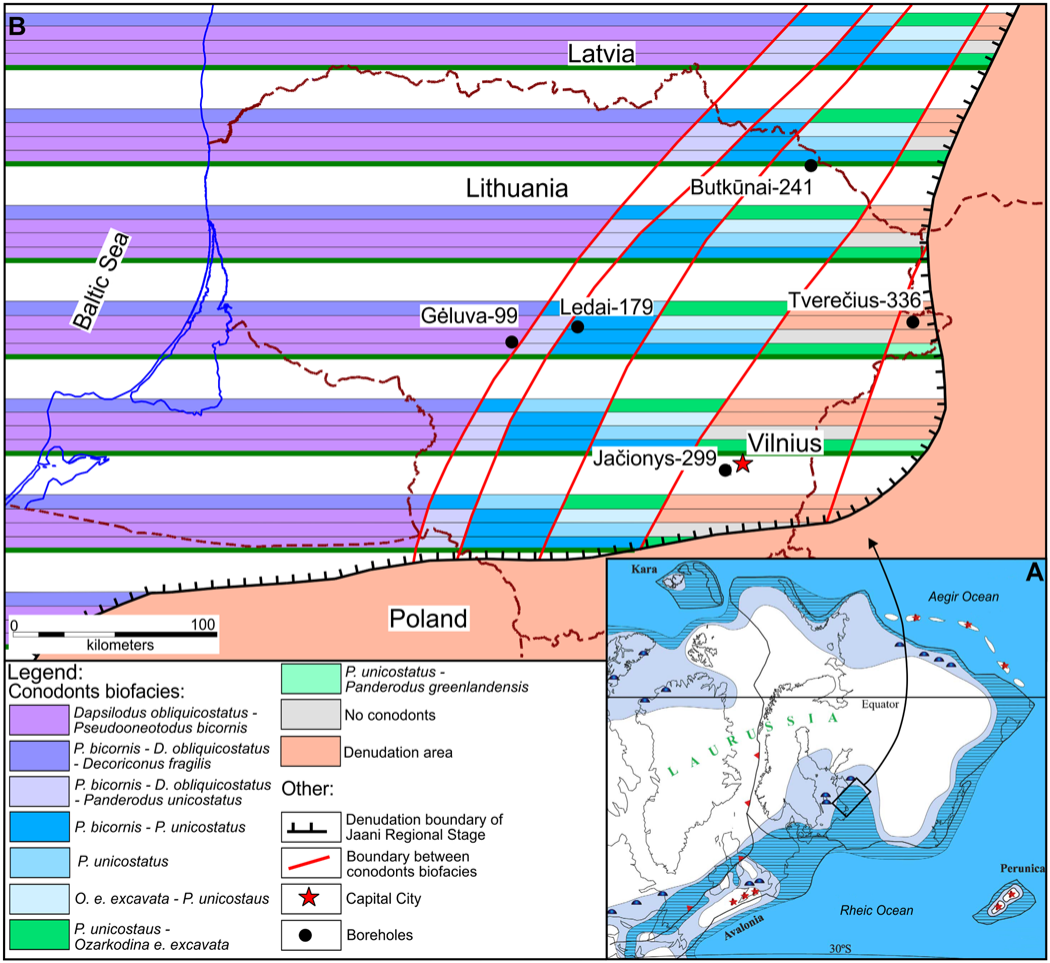
Geographic and paleogeographic locations of deep cores used in the analysis. (A) Map of Laurussia (Euramerica) during the Wenlock age (~430 Ma) (Modified from the [26]). (B) The distribution of deep cores in conodont biofacies during the Jaani regional stage (modified from [51]). Vertical, stripe-like biofacies succession at each point represents temporal species assemblage development.

In this study, we analyze the degree to which abundance, temporal structure as measured by a set of parameters, extirpation rates (local extinction rates), colonization rates and habitat preferences of conodont species, as estimated using geophysical proxies, determined conodont species odds of survival during the mid-early Silurian mass extinction event. The object of our study is the uppermost Llandovery and lowermost Wenlock conodonts of the Lithuanian section of the Silurian Baltic basin (Fig 1). Bivariate logistic regression models and also the model selection procedure of multivariable logistic regressions revealed that the best model for describing extinction risk includes water depth proxies and abundance distribution skewness. Similar results were found from so-called regression tree analyses, which revealed hierarchical association of predictors of extinction risk with species survival.

## Materials and Methods

### Geological Setting

All of the data on conodont element abundances and environmental proxies that were used in the statistical analyses of this study were gathered from deep-core material from the five Lithuanian locations. During the Silurian period, the studied territory formed part of an epicontinental sea, the so-called Silurian Baltic basin, composing the southeastern section along the shoreline. This basin was located on the Baltica paleocontinent (Fig 1a). During the early Silurian period it was situated in tropical and subtropical latitudes south of the equator [26]. The strata that were investigated in this study for conodont abundance were formed during the late Llandovery (Telychian) and early Wenlock (Sheinwoodian) stages of the early Silurian period [27,28]. The Ireviken event occurred at the boundary of those two stages and is thus approximately 433.4 million years old [29]. This interval corresponds to two generalized conodont biozones: the *Pterospathodus amorphognathoides* and *Kockelella ranuliformis* zones [29, 30].

**Fig 2:**
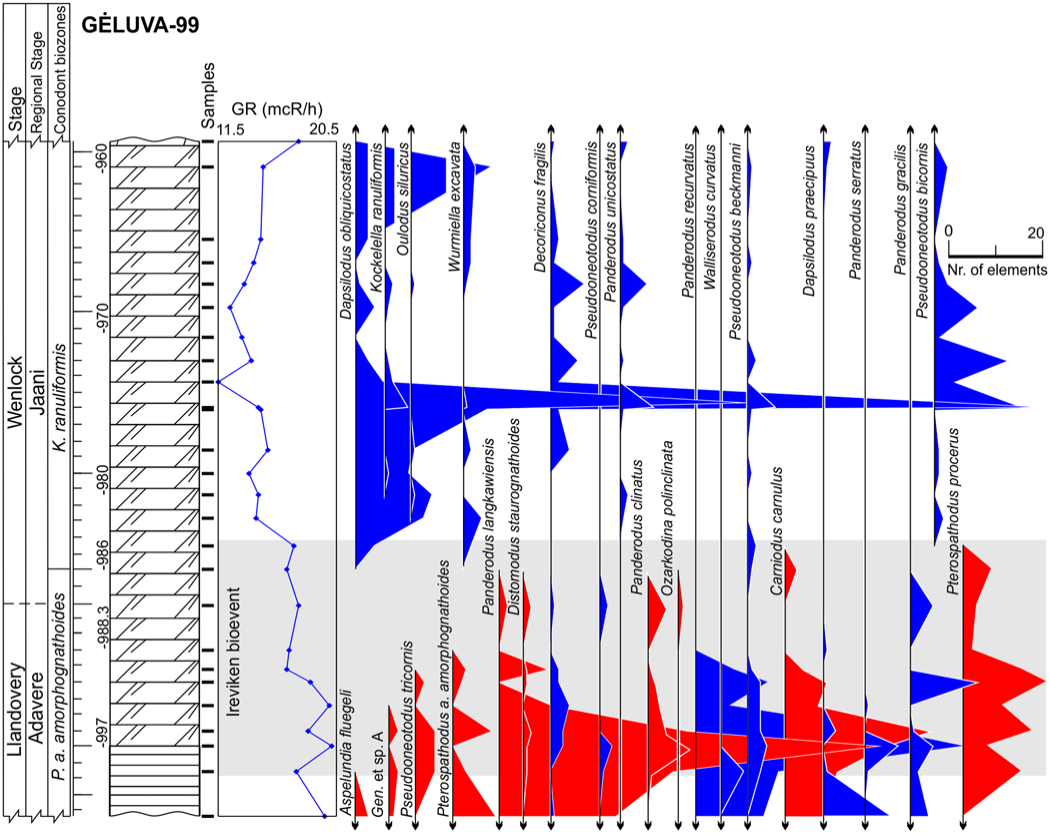
Lithology, gamma-ray logs and abundance time series of conodonts in the Gėluva-99 core. Surviving species colored in blue, victims are colored in red. Lithology legend in Fig 6.

**Fig 3:**
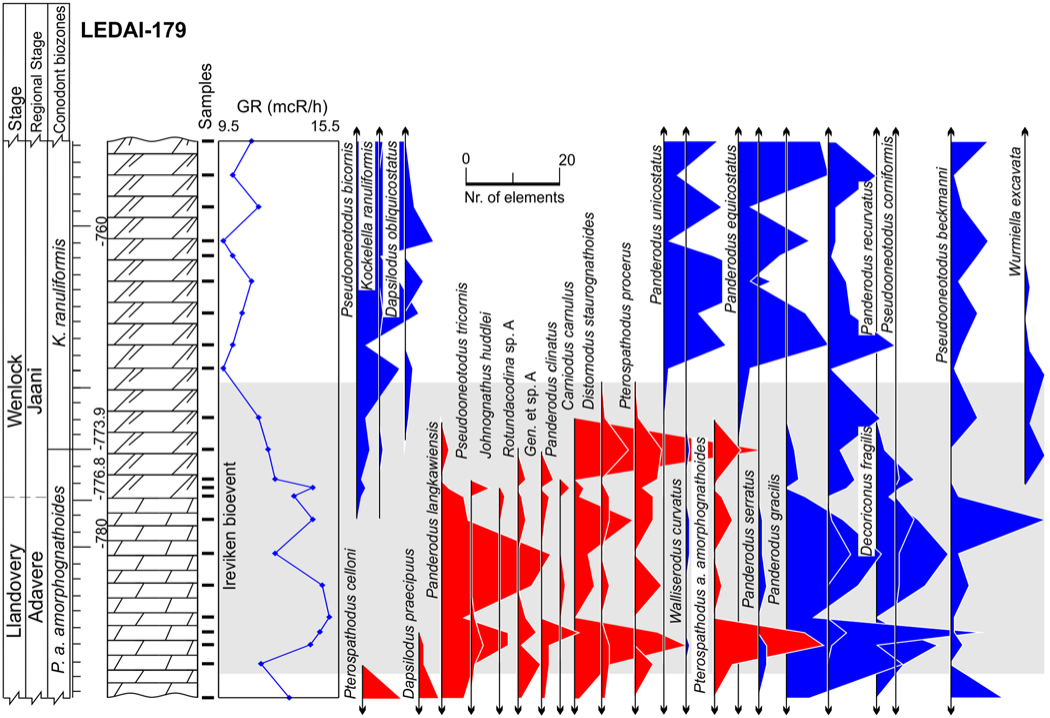
Lithology, gamma-ray logs and abundance time series of conodonts in the Ledai-179 core. Surviving species colored in blue, victims are colored in red. Lithology legend in Fig 6.

**Fig 4:**
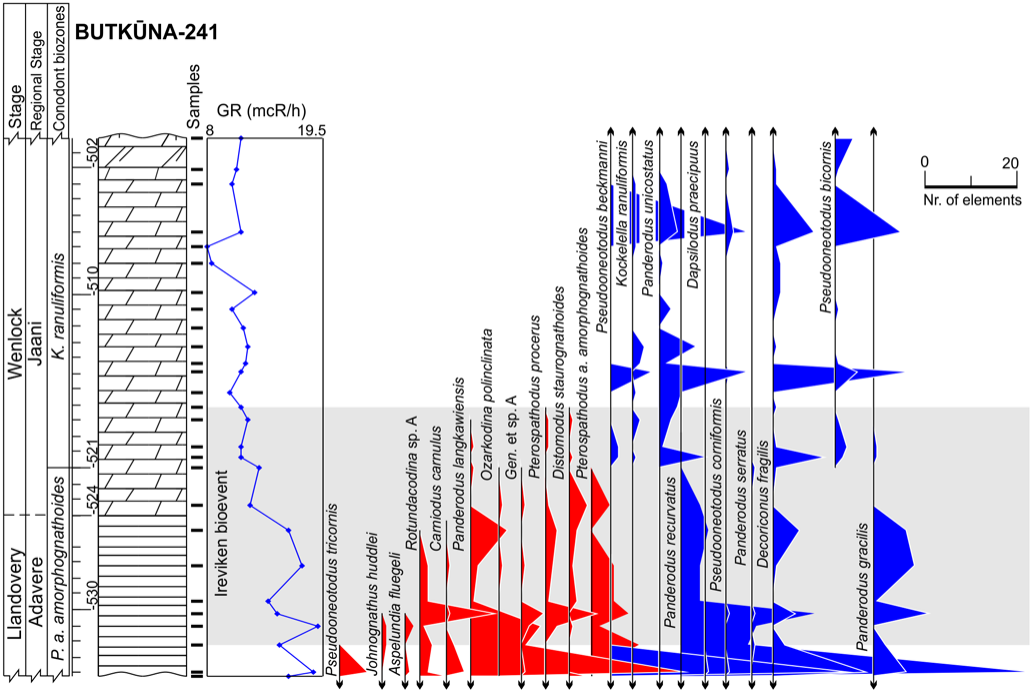
Lithology, gamma-ray logs and abundance time series of conodonts in the Butkūnai-241 core. Surviving species colored in blue, victims are colored in red. Lithology legend in Fig 6.

**Fig 5:**
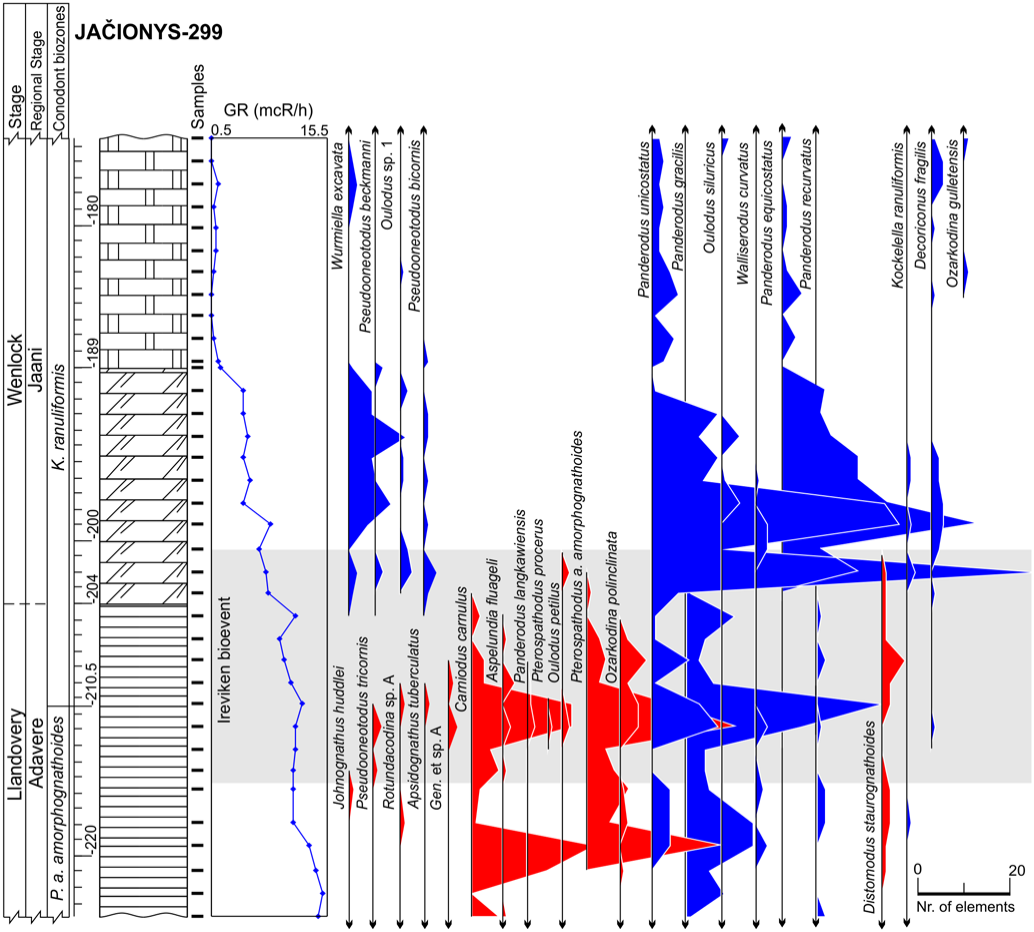
Lithology, gamma-ray logs and abundance time series of conodonts in the Jačionys-299 core. Surviving species colored in blue, victims are colored in red. Lithology legend in Fig 6.

**Fig 6:**
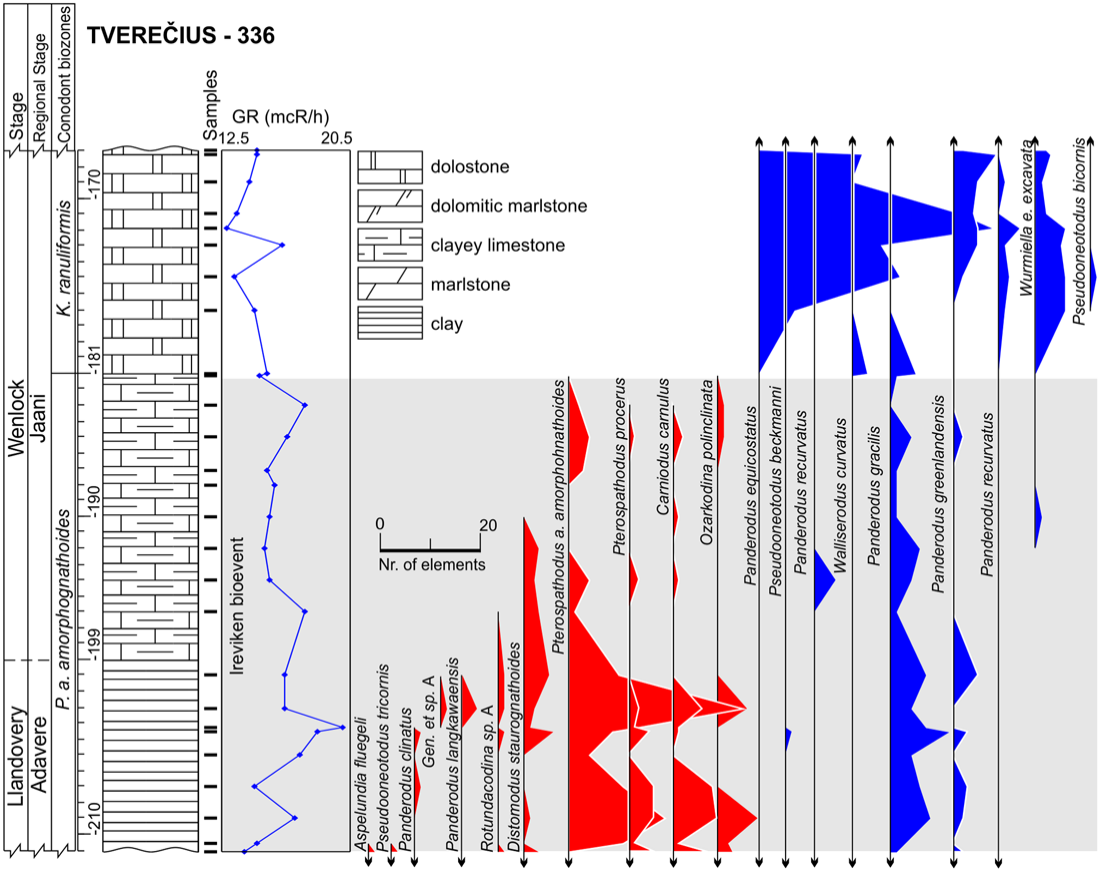
Lithology, gamma-ray logs and abundance time series of conodonts in the Tverečius-336 core. Surviving species colored in blue, victims are colored in red.

**Fig 7:**
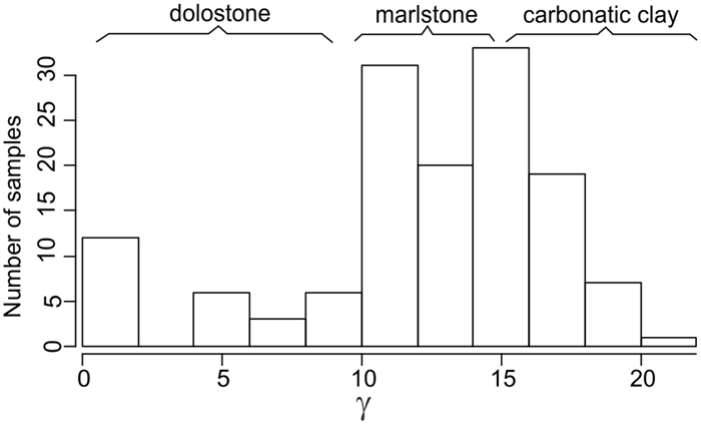
Frequency distribution of samples as a function of values of environmental proxy (gamma-ray intensities at sampling sites ≈ amount of clayey and organic material in a rock).

Five deep cores (Gėluva-99 (26 samples), Ledai-179 (22 samples), Butkūnai-241 (27 samples), Tverečius-362 (27 samples), and Jačionys-299 (36 samples)) were collected as samples of all of the paleoenvironmental belts in the basin, from coastal, lagoonal carbonates to deep-water, clayey mudstones (Fig 1b, Figs 2–6, and Fig 7). All studied cores spanned the same sequence stratigraphic system tracts which were constrained using conodont biostratigraphy and lithological information. Temporal resolution, though varying, is comparable between different core sections. Shortest (measuring in rock thickness) core section covers 33 m and the longest spans 49 m of strata (Figs 2–6). This sampling design ensured the inclusion of a wide range of specialized conodont species and at the same time allowed us to evaluate the influence of environmental preferences on extinction risk.

### Sampling

In this study, we examined conodont element abundance records for the samples, which were prepared in the late eighties [30]. At the time of preparation, sample weights were not recorded. However, the sample sizes were kept in the same range (300 to 400 g) so that the abundance sampling variation remained random with respect to sample position in the stratigraphic column, geographic locality, and conodont species. The random variation in sample sizes may have introduced noise and lower autocorrelation values. However, species abundance parameter estimates used in this study were drawn from the same data set. Hence, value differences between species are not biased and instead represent a genuine primary signal. Because conodont elemenets were likely retained throughout individual’s life [31], their numbers could be used as direct proxies of organismal abundance. On the other hand, it could be argued that different conodont species may have possessed varying numbers of elements, which may distort interspecific comparisons of numerical abundance. However, most of the clades documented in the natural assemblages of conodont elements possessed exactly 15 elements in their apparatuses [32]. Thus, as an initial approximation, our assumption of interspecific comparability of conodont abundance using element counts should be valid.

The conodont element samples were extracted from the rock matrix using 10% weak-buffered acids: acetic acid for rocks with limestone cement, and formic acid for rocks with dolomitic cement. Additionally, if rocks contained large quantities of binding, non-dissolvable organic material, samples were treated with the hydrogen peroxide. Residue was then dried and strained through sieves of several different diameters. Conodont elements were handpicked through an exhaustive examination of residue fractions.

Overall, we gathered abundance information on 31 formally described conodont species. Additionally, in the species set, there were three, formally undescribed species (Gen. et sp. A; Oulodus sp. 1; and Rotundacodina sp. A), which were also used in extinction selectivity calculations. For the purposes of the analysis, we categorized species that had gone extinct during the Ireviken event as “0” and those that survived as “1” (see Supplementary Material) based on the taxonomic compilations of Jeppsson [4,5] and based our own observations of stratigraphic lineage distribution in deep cores.

### Predictors of species extinction risk

In order to evaluate selectivity of conodont species extinctions during the Ireviken event we evaluated effects of ten parameters derived from conodont abundance and gamma-ray time series. In this study, we measured average species abundance levels ($\overset{-}{a}$) in their stratigraphic ranges by counting numbers of conodont elements in samples from all of the deep cores (stratigraphic sections) of non-zero, focal species abundance, omitting zero values that were occasionally observed in the ranges. In addition, we measured levels of species abundance variability. However, it is widely recognized that this property is notoriously difficult to meaningfully compare when using time series of different lengths [33, 34], which is to be expected if certain species went extinct at earlier times than others. This follows from the fact that auto-correlated time series, which are often used for abundance counts, are prone to a mathematical artifact called “infrared catastrophe” [35], which results in an indefinite growth of variance when time approaches infinity. Hence, simple abundance variance comparisons are meaningless. One more complicating factor is that in paleoecological time series of conodonts, abundance variability is proportional to average abundance (Taylor’s law [36, 37]). For example, the relationship between localized (three samples excluding zeros) variance and abundance averages for the species *Panderodus unicostatus* as observed in the Jačionys—299 section follows a power law (*σ* ^2^(*a*) = 0.48 * *a* ^1.2^, *r* = 0.76, *p* = 0.004, *n* = 28). Hence, to compare different species and factor out the influence of absolute abundance, we used a modification of the variation coefficient as a parameter. Additionally, to factor out another possible element of bias—the influence of time series lengths—we calculated local variation coefficient averages. Each variation coefficient was counted from two nearby, non-zero samples, which ensured comparisons over the smallest possible time scales that were identical (tens of thousands of years). The formula for the averaged variation coefficient calculation ($\overset{-}{CV}$) from the localized variation coefficient is:

$$\bar{CV}=\frac{\sum_{k=1}^{k=j}\sum_{i=1}^{i=b}CV2_{ik}}{n}$$

Where $\overset{-}{CV}$ is the averaged variation coefficient, *CV2* is a two sample localized variation coefficient, *i* is an index of the local variation coefficient in a given stratigraphic section, *b* is the last variation coefficient in a section, *j* is the number of stratigraphic sections, k is an index of a section, and *n* is the overall number of local variation coefficient calculated for a given species and summed over all sections.

As the structure of abundance variation is highly complicated, we used additional measures that yielded complementary information on the anatomy, i.e., temporal dependencies in time series abundance values and abundance distribution shapes. As measures of long-term memory, we calculated so-called Hurst exponents, which proved useful in describing variability features of modern populations and in providing variance estimates [38], as well as slopes of log-power against estimated power spectra fluctuation log frequencies. To better evaluate the time series, for the calculations of both measures, we used the longest stratigraphic sequences (in sample numbers) of the focal species without omitting zero values (because this could diminish long-term variations). The Hurst exponent (*H*) was calculated, on a demeaned stratigraphic series of abundance using the expression given by Pfaff ([39]; p. 67):

$$\left(\frac{R}{S}\right)\;=\;\frac{1}{S_{T}}\;\left[max_{1\leq k\leq T}\;\sum_{j=1}^{k}\left(a_{j}\;-\;\bar{a}\right)\;-\;min_{1\leq k\leq T}\sum_{j=1}^{k}\left(a_{j}\;-\;\bar{a}\right)\right]$$

Subsequently,

$$H=\frac{\text{log}\left(\frac{R}{S}\right)}{\text{log}\left(T\right)}$$

Here, ($\frac{R}{S})$ is a rescalled range statistic, H is a Hurst exponent, j is a sample index in a stratigraphic series, ST is a maximum likelihood standard deviation estimator of abundance in a time series, and T is a time series length based on the number of samples. Hurst exponent calculations were executed using R computing software [40].

Power spectra, which were used in the scaling exponent calculations, were calculated using the Lomb method, which is suited to stratigraphic sequences with variable sampling intervals [41], using the PAST statistical package [42]. Scaling exponents (ν) of the power spectra were calculated as minus regression slopes of ordinary least square regressions of power natural logarithms against natural frequency logarithms [34]. To avoid exaggerating the effects of logarithms on small numbers, we added a constant (“1”) to all of the spectra frequency and power values prior to the slope calculations. Scaling exponents were calculated for the longest records (in numbers of samples) of stratigraphic series for a given species, including all of the samples between the first and last appearances.

It is known that species are characterized by highly skewed distributions of local numerical individual abundance [23,43]. This was also the case for the conodont species that we studied. Differences in the degree of skewness may reflect species tendencies to achieve abnormal numbers, which may act as an important survival feature. Large positive excursions may be beneficial to species survival, and a capacity to occasionally reach very low numbers may be related to extinction risk. Hence, in our analysis, we estimated this property as a skewness of the species abundance distribution, excluding all zero observations *G*(*a*).

In addition to measures of abundance and variability structure, we estimated, as predictors of global extinction, rates of local species extirpation and colonization. We adapted measures developed by Legendre and others [44] for the calculation of short-term metapopulation dynamical processes to the paleoecological context. Our method differs in that we estimated parameters from apparent stratigraphic ranges while excluding first and last species appearances in given sections, following methods of stratigraphic record completeness measurement via “gap analysis” [45]. This estimation method functions in the following manner (using notations by Legendre and others [44]). Suppose that there is a time series (excluding first and last appearances) of abundances XX000X0XX, in which “X” denotes nonzero abundance while “0” represents zero abundance of a given species in a sample. We then calculate the length of the time interval (in number of samples) to the last sample in the analyzed sequence as *t*, element abundances in samples as *n*(*t*), the number of zeros in a series as *z*(*t*), the number of local extinction (extirpation) events up to time *t* as *ε*(*t*), and *i*(*t*) as the number of colonization events up to time *t*. The apparent rate of extirpation (*E*(*t*)) is estimated using the following formula:

$$E(t)=(\begin{array}{l} \frac{\varepsilon (t)}{t+1-z(t)}\text{ if }n(t)=0\\ \frac{\varepsilon (t)}{t-z(t)}\text{ if }n(t)>0 \end{array}$$

The rate of apparent colonization (*I*(*t*)) is measured in following manner:

$$I(t)=(\begin{array}{l} \frac{i(t)}{z(t)-1}\text{ if }n(t)=0\\ \frac{i(t)}{z(t)}\text{ if }n(t)>0 \end{array}$$

In the presented example, the time interval length until time *t* is equal to“8,” the number of zeroes is equal to “4,” the number of extirpation events is equal to “2,” and there are two colonization events. In this case, the extirpation and colonization rates are both equal to “2/4.” Apparent extirpation and apparent colonization rate calculations for long-term abundance changes were also calculated for the longest (measured in numbers of samples) stratigraphic series of each given species. In this way, we ensured the highest completeness values and the adequacy of stratigraphic information.

The third set of parameters used in the predictions of conodont species extinction risk are related to substrate preferences, as reflected by geophysical information. We employed gamma-ray radiation intensity techniques in the boreholes, which were calibrated to core sampling depths. Gamma rays in the sedimentary rocks are primarily emitted through radioactive isotope fission of K, U, Th. These chemical elements are typically concentrated over distinct, mineral and organic matter phases. K and Th are typically located in detrital minerals, and U is typically concentrated in clayey minerals and organic detritus [46,47]. Hence, the gamma radiation intensity should be proportional to rock phase concentrations. Clayey and biodetrital organic materials are often found in sediments that form at greater water depths in mixed carbonatic-siliciclastic Lithuanian geological sections of lower Silurian time. Here in coastal (shallow water) environment carbonates constitute the dominant sediment form [28]. This proxy can thus be used as a first order approximation of water depth at which rock-forming sediments accumulate in the basin (larger gamma radiation values correlate with sediment formation at greater water depths). Turbidity currents and other local local factors of clastic sedimentation most probably played a minor role inducing high frequency noise to long term sedimentary trends. Cyclostratigraphic analyses of Western Lithuanian upper Wenlock sequences have revealed that gamma ray proxies accurately reflect eustatic sea level changes documented in other places by other means [29,48], with smaller gamma radiation values documented in the intervals of low-stand system tracts. Similarly, in the present case it could be seen (Figs 2–6) that during the Llandovery-Wenlock transition there is consistent drop in gamma ray intensities, which is most probably related to the widely recognized early Sheinwoodian drop in the eustatic sea level [49,50]. The *in situ* origin of conodont elements in rock samples is also confirmed by their excellent level of preservation. Rounded and abraded elements are rare.

For each conodont species and using gamma-ray proxies, we calculated three metrics—average gamma radiation values at positive (non-zero count) sample depths ($\overset{-}{\gamma }$), standard deviation (*σ*(*γ*)), and gamma radiation intensity skewness (*G*(*γ*)) at positive sample depths. The first (average) metric represented species preferred water depths. Standard deviations accordingly represented a range of occupied water depths, and skewness represented distribution shapes over water depth gradients. While it is still debatable whether conodonts are truly related to substratum (nektic vs. nekto-benthic controversy) conditions, distinct patterns of paleoenvironmental distribution among these species are related to water depth gradients, which are important features of community ecology [51].

### Statistical techniques for extinction selectivity detection

In an effort to estimate extinction selectivity values during the Ireviken event, due to the binomial structure of the response function (“0” if species went extinct, and “1” if species survived the event), we employed Generalized Linear Modeling (GLM) using logistic regression techniques via the logit link function [52]. Before constructing and comparing complex, multiple logistic regression models, we tested simple bivariate regression patterns. After evaluating all ten factors separately, those which exhibited a slope describing parameters β (log-odds) that were found to be statistically significant in predicting extinction risk at the p<0.1 level were selected for further analysis. In order to measure effect sizes of different variables of conodont extinction risk, we used Tjur’s coefficient of discrimination (D) for logistic models which is a measure analogous in its properties to r^2^ of least square regression models [53]. Discrimination coefficients were calculated in R environment using package ‘binomTools’ [54]. Multiple explanatory variable models were constructed as an exhaustive comparison of all combinations of the best predictors. The complex models were compared and judged on the basis of goodness of fit and complexity, as measured by a number of parameters, using the information theoretical model selection approach [55]. Due to the limited number of observations we used corrected Akaike information criterion (AICc) and computed associated Akaike weights (ω) using R programming software provided through the “AICcmodavg” package [56]. Parameters of the best multivariate logistic model were accepted as significant if they predicted extinction risk at p<0.05 level. Because multiple regressions must be carried out with a complete data set (there should be no missing values), an increase in the number of variables causes reduction in the total number of observations. Hence, the bivariate and multivariate regressions are not directly comparable due to the presence of the varying information completeness. However, major patterns consistent with both model sets were detected.

To test the robustness of the logistic regression results, we performed additional analyses using the machine-learning Classification and Regression Trees (CART) methodology. It can decipher non-linear, hierarchical, context-dependent interactions between explanatory variables [57, 58]. We implemented the procedure using the R package “rpart” of the R computing environment [59]. As a measure of node impurity, which was utilized in evaluating regression tree splitting points, we used the ANOVA metric, as it allows gathering of information on classification errors. The only observations that were discarded from the calculations were those that were not recorded for particular variables, thus maximizing the utility of available data [59]. While evaluating the predictors, we used default package settings.

## Results

**Fig 8:**
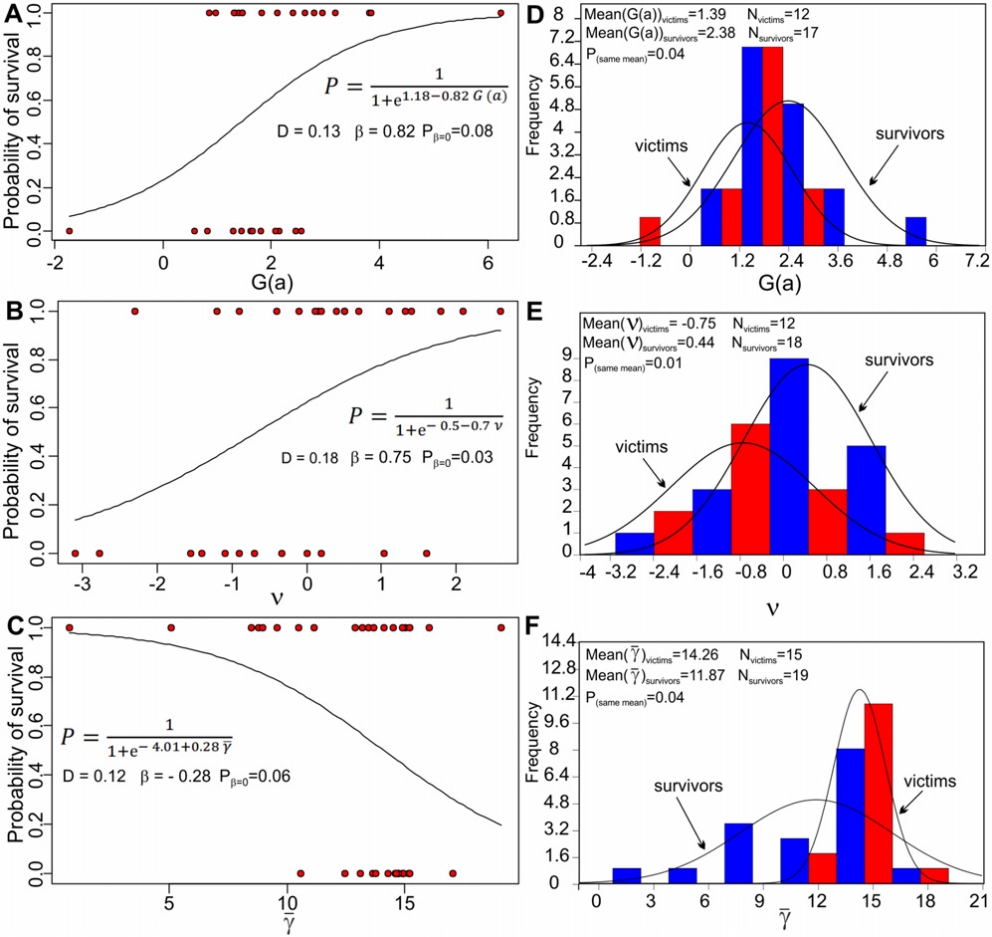
Logistic regression models that include the best predictors of conodont survival during the Ireviken event. (A) Skewness of species abundance distributions (*G*(*a*)); (B) Spectral exponents (ν) calculated from the longest stratigraphic abundance series for a given species; (C) Average gamma-ray values ($\overset{-}{\gamma }$) at the positive sample sites for a given species. Equation abbreviations: P, probability of survival, β, logistic variable regression coefficient (log-odds of survival), P_β = 0,_ the probability that coefficient is β equal to zero. Frequency distributions (E-F) of victims and survivors with estimates of their average values of predictors and the results of Student’s t-test for equality of means: (E) Skewness of species abundance distributions (*G*(*a*)); (F) Spectral exponents (ν); Average gamma-ray values ($\overset{-}{\gamma }$) at the positive sample sites.

The bivariate logistic regression study of ten predictors of conodont extinction risk during the Ireviken event reveals several interesting patterns. The average abundance (β = 0.06; p = 0.51; n = 34; D = 0.01), averaged variation coefficient (β = 0.39; p = 0.86; n = 31; D = 0.008), Hurst exponent (β = 6.49; p = 0.2; n = 30; D = 0.05), extirpation rate (β = 1.02; p = 0.32; n = 29; D = 0.03), colonization rate (β = 2.12; p = 0.26; n = 27; D = 0.04), environmental proxy standard deviation (β = 0.31; p = 0.39; n = 31; D = 0.02), and skewness degrees and direction *G*(*γ*) values of environmental preference (β = - 1.21; p = 0.11; n = 30; D = 0.09) were statistically insignificant (at p<0.1 level) as descriptors of extinction risk. The best descriptors (Fig 8) found through the bivariate logistic regression analyses included scaling exponent values (ν) of power spectra (β = 0.75; p = 0.03; n = 30; D = 0.18), followed by average substrate preferences ($\overset{-}{\gamma }$) as described by gamma-ray proxies (β = - 0.28; p = 0.06; n = 34; D = 0.12) and skewness *G*(*a*) of local species abundance (β = 0.82; p = 0.08; n = 29; D = 0.13).

From this group of three significant descriptors of conodont species extinction risk, we constructed an exhaustive set of complex models: three with two predictors and one with all three predictors. Those models were constructed from the culled data set, in which species possessed all of the parameters of concern (overall n = 29 observations). The ranking of models based on the AICc and calculated Akaike weights (ω) revealed that the best model is one of the simplest. It included explanatory variables of species abundance distribution skewness (*G*(*a*)) and average substrate preference $\overset{-}{\gamma }$ (Tables 1 and 2). Both variables in the most effective complex model were statistically significant at the p<0.05 level. It was found that larger species abundance distribution skewness and smaller rock gamma-ray values in rocks in which species were found (i.e., carbonatic, shoreline environments) correlate with a higher probability of conodont species survival during the Ireviken event. Another variable (ν), as revealed by the model selection procedure, does not substantially explain deviance once added to the model which also includes abundance distribution skewness and ranked lowest among models (Table 1). However, the correlation between (*G*(*a*)) and (ν) is very low and insignificant (*r* = 0.12, *p* = 0.15, *n* = 29). Hence, the reason why these variables are redundant is not easily explainable.

**Table 1:** A comparison of complex logistic models (based on n = 29 observations) of conodont species survival probability during the Ireviken event. Models are compared and ranked based on AICc and associated Akaike weight (ω) values.

Model	Number of parameters	AICc	ω	Log-likelihood	Deviance explained (%)	Tjur’s coefficient of discrimination (D)
*G*(*a*), $\overset{-}{\gamma }$	3	27.05	0.71	- 10.04	48.9	0.51
*G*(*a*), ν, $\overset{-}{\gamma }$	4	28.91	0.28	- 9.62	51.0	0.52
ν, $\overset{-}{\gamma }$	3	36.80	0.01	- 14.92	24.1	0.36
*G*(*a*), ν	3	36.88	0.01	- 14.96	23.9	0.25

**Table 2:** Statistical estimates of the best multivariable logistic model.

Variable	β*i*	SE	*p*
*G*(*a*)	2.41	1.11	0.03
$\overset{-}{\gamma }$	-1.09	0.50	0.02

**Fig 9:**
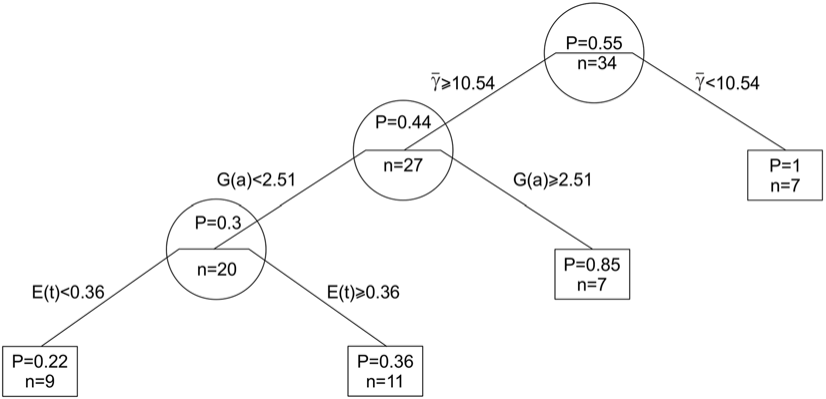
Regression tree model revealing factors associated with conodont species survival during the Ireviken event. Estimated probabilities of survival (P) and corresponding numbers of species (n) at each node are positioned in circular arrangements, and corresponding survival probabilities and species numbers at terminal branch tips form square-shaped arrangements. Symbol abbreviations: ($\overset{-}{\gamma }$) average gamma ray values at the positive sample sites, (*G*(*a*)) skewnesses of species abundance distribution, (*E*(*t*)) apparent extirpation rates.

The regression tree analysis of conodont species survival reveals similar results as the model selection procedure of the best predictors. The most significant predictors of extinction, according to this analysis, are average gamma-ray values ($\overset{-}{\gamma }$), species abundance distribution skewness (*G*(*a*)), and extirpation rates (*E*(*t*)) (Fig 9). Species that lived in shoreline environments (gamma-ray intensity <10.54 μR/h), as predicted, exhibited a 100% survival probability rate. Further, the probability of survival for species that lived in deeper environments was influenced by structures of abundance dynamics as reflected by (*G*(*a*)). Higher values of abundance skewness (> 2.51) in deeper environments were associated with a higher probability of survival (85%). Species found in deeper environments which exhibited low degrees of abundance distribution skewness (<2.51) had a 30% probability of survival. Within this trait group (lower skewness and deep water environments), an important factor that emerged was the apparent local extirpation rate (*E*(*t*)), which was unexpectedly positively related to survival probability. Species with rate values higher or equal to 0.36 demonstrated a survival probability of 36%, and species with *E*(*t*) values lower than 0.36 exhibited the lowest probability of survival (22%).

The regression tree model less accurately predicts species survival (it explained 40.8% of deviance) than the best multiple regression model, which explained 48.9% of species extinction deviance. However, this discrepancy may have emerged because the regression tree utilized all of the data points while the multivariable logistic regression model used a subset of the data.

## Discussion

The results of the logistic regression analyses show that there is no statistically significant association between average local conodont abundance and extinction risk during the Ireviken event. This pattern is congruent with the results of previous paleobiological macroevolutionary studies, which largely revealed relatively weak to non-existent [60, 61, 62] (including cases of mass extinction [63]) and occasionally nonlinear and context-dependent influences [64, 65] of local abundance on extinction probability. However, it is possible that these patterns reflect weak statistical testing methods, as small abundance effect sizes (and other statistically insignificant factors) on risk were confounded by small sample sizes used in the analyses [66]. Another possible factor which could influence apparent abundance patterns (and their effects on extinction selectivity) in the fossil record is time averaging. It increases temporal autocorrelation of time series [67] and also changes shapes of species rank abundance distribution (RADs) by increasing the proportion of rare species [68]. It is unlikely that temporal mixing by means of bioturbation affected autocorrelational properties of time series, because samples in analyzed time series were spaced far apart of each other (on the order of ≈1.5 m). However, postmortem changes in relative abundances of species, due to temporal averaging should have had some randomizing (signal destroying) effect on the apparent role of abundance in extinction selectivity of conodonts.

It was suggested and affirmed by modern ecological records that population size variability may be a significant factor that increases extinction risk [33, 34, 35, 69]. The theoretical explanation for this trend is that reproduction, drives organismal abundance, is a multiplicative process, and variation decreases the quantity of multiplicative product, which may be envisioned as the fitness of a given population of types [70]. However, our study revealed that local variability over the shortest conceivable time scale (tens of thousands of years) is positively related to survival despite the fact that the slope probability equal to zero is very high (nearly approaching unity). Hence, this value does not have any statistical significance. This lack of relationship between local abundance variation levels and extinction risk could be explained by Steven Stanley’s proposal ([71], p.198) that the magnitude of abundance fluctuations may increase stochastic extinction risks for incipient species, peripheral isolates, or species that have been heavily reduced in abundance due to other factors. However, this would not apply for fully established and widespread lineages, for which complex geographic structures buffer against localized random “shocks”.

However, complex patterns of abundance variability, as reflected by the other two parameters ((ν) and (*G*(*a*))), are significantly associated with species extinction risk. The spectral exponent value (ν), which reflects the “color” of a noise (degree of its autocorrelation), is the most significant predictor of extinction risk according to simple bivariate logistic models. It has been shown in previous empirical studies based on the studies of modern organisms that noise color is a significant determinant of population extinction risk [34]. Theoretical expectations are more complicated and contingent on dynamic model specifications, and especially in relation to the mode of frequency regulation, e.g., if it is under- or over-compensatory [35, 72, 73, 74]. However, these explanations generally predict lower, long-term extinction risk for those populations that are characterized by reddened dynamics [35,75]. The greatest survival chances associated with populations related to black (ν>2) noises [76]. Hence, our findings confirm the positive effects of temporal autocorrelation on abundance change in the survival of higher (species) level evolutionary entities during this episode of mass extinction. However, based on the context of other results presented in this study, it is difficult to determine whether this quality is causally related to the species’ survival. Model selection procedure of multivariable logistic models (Table 1 and 2), suggest that this variable is informationally redundant with skewness of abundance distribution and also average gamma ray values.

In addition, it is interesting that the higher spectral exponent values (ν) were negatively related not just to species extinction risk but also to local extirpation rates (*r* = -0.33, *p* = 0.07, *n* = 30). These values were also positively, though insignificantly, correlated with colonization rates (*r* = 0.28, *p* = 0.14, *n* = 27). Possibly due to small effect sizes, these lower-level, spatially localized processes were found through the bivariate logistic analyses to be insignificant in determining global species extinction risk. Moreover, as was revealed by the same analyses, both extirpation and colonization rates were positively associated with survival probability. In addition, the inferred regression tree structure confirmed this relationship by revealing the localized context of extirpation rate action. Higher extirpation rates were positively related to the survival of species exhibiting higher levels of extinction risk (Fig 9). This suggests that species with elevated spatiotemporal turnover rates may have a higher probability of survival. This inference would complement scale transition theories concerning metacommunity dynamics, which state that species organismal fitness variability between communities in a metacommunity network can positively affect the survivorship of individual organisms of a species while generating qualitatively different dynamics in comparison to spatially homogenous “mean field” models [77, 78,79]. In any case, it appears that there is no simple relationship between lower- and higher-level processes, as was suggested in earlier studies on mass extinctions [79].

It is surprising, however, that the Hurst exponent (*H*) does not show a statistically significant association with survival probability (though it is fairly close to the minimal levels of significance *p* = 0.2). On theoretical grounds, this metric measures the same property as (ν)—the level of autocorrelation in a time series. Despite being insignificant, its positive association with survival is the same as that of spectral exponents, which illustrates the prevalence of primary signal in the data. Hence, this situation likely reflects weaker qualities of (*H*) as a statistical predictor. This may be attributable to the fact that we employed the Lomb method during spectra estimations (which is suited to unevenly spaced samples in a stratigraphic series) and then applied linear regression during the estimation of (ν) (which utilizes all data points of a power spectrum). On the other hand, (*H*) values are estimated from two extreme points in a time series, which are later normalized for variance and sequence length. Thus, this discrepancy in predicting outcomes is likely related to the lower degree of accuracy and precision generated by Hurst estimates in noisy, stratigraphic, paleobiological contexts.

The skewness of abundance distribution emerged as an important predictor of survival in both the bivariate and multivariable regressions as well as through the regression tree analysis. The multimodel selection analysis showed that the best model for describing survival includes (*G*(*a*)) and ($\overset{-}{\gamma }$) while the least effective model includes (*G*(*a*)) and (ν). This reveals the redundancy of information between these latter variables (Table 1) and the informative capacities of (*G*(*a*)).

Large values of positive abundance distribution skewness reflect the propensity of species to reach extreme abundance levels relative to typical figures. It has often been observed that certain species in fossil records exhibit a tendency to reach extreme numbers and then quickly return to their typical abundance values. It was also suggested by Anthony Hallam [80] that such species tend to be *r* selected, non-equilibrial, and opportunistic species. If this is true, this may provide insight into the dynamics of ecosystem processes that occur during mass extinction events. Rather, both during and shortly after mass extinction events due to a loss of species and functional ecosystem diversity, dynamics of so-called flux ecosystems experience larger fluctuations [81], as recorded in studies of Triassic and Jurassic ammonoids [82]. Given these ecosystem dynamics, possible mechanisms of selective survival for quickly reproducing species could be revealed by applying Richard Levin’s permanent nonequilibrium coexistence model as elaborated by Loreau [83], which is similar to Valentine’s [84] conceptual model of fluctuating resource subdivisions between generalist and specialist species. The model states that ecologically similar species may coexist due to differences in the shapes of their population growth curves and variations in consumed resource quantities. According to Loreau [83], linearly increasing species consume resource “averages” while nonlinear species consume resource “variances.” Hence, if disruptions to normal ecosystem functioning followed by an increase in resource production variance occur during an extinction event, opportunistic species with more nonlinear growth curves would possess an abundance edge over their competitors These dynamic patterns may translate into a higher probability of survival both during and shortly after an episode of mass extinction. Though, in order for links between variation patterns and species survival during mass extinction events to be confirmed, more fine-grained data are needed which will include proxies of biological productivity.

The proposed mechanism of survival for high *G*(*a*) species is not the only possible explanation. Rapid and unchecked growth (due to low population densities) after environmental stress amelioration or new environment invasion significantly affects long-term ecological dynamics, as documented in quaternary palynological fossil records [85]. This phenomenon, which is occasionally referred to as “waves of life” considerably affects geographic evolutionary processes [86]. It may enrich “exaptive pools” [87, 88] of genotypic and phenotypic variation, which may later play a major role in increasing evolvability levels during transient periods after perturbation. In addition to overall exaptive variation accumulation, it is possible, as was suggested in previous studies, that during initial expansion processes, certain phenotypic traits that accelerated the dispersal of individuals grew more concentrated, resulting in what is called “spatial sorting” of variation [89]. This process may phenotypically strengthen species capacities to invade new environments to an even greater degree possible in equilibrial states of maximal organismal fitness. Thus, species with higher levels of *G*(*a*) are more likely to possess dynamic eco-evolutionary qualities that may improve species level fitness due to an increase and associated structuring of phenotypic variation.

If the above listed mechanisms were indeed important and can explain positive relations between abundance skewness and conodont survival during the Ireviken extinction event, this implies the occurrence of both narrow and broad species selection due to survival mechanisms based on species-level, ontologically emergent properties and bottom-up, ‘aggregate,’ organism-level selective effects (sensu [90, 91]). This is true because the first explanation, as discussed in this paper, highlights the importance of organismic-level life-history traits in mediating degrees of population growth non-linearity. Another explanation states that species survival was also based on phenotypic variability levels (which are typically viewed as species level traits [92, 93]) at the time of mass extinction, controlled by specific population dynamics. However, in addition to these explanations, other explanations of fluctuation patterns may be related to differences in *G*(*a*) levels. Rather, a species capacity to experience different oscillation levels over geological time scales is also tied to the species spatial structure. This may imply that processes occur over different time scale at several (mostly higher) organizational levels [94]: local avatar (populations of ecologically interacting individuals [95]) dynamics and interactions from changes in regional and global biota. Thus, differences in fluctuation patterns among paleoecological abundance time series representing intervals up to hundreds of thousands of years long emerge as a result of reciprocal relations at the species level and community and organismic traits such as spatial range, range structures, metapopulation “wiring” [94] and metacommunity networks [96]. However, to fully correlate the observed regularities with the mechanistic explanations, additional studies must link demographic and phenotypic predictions of proposed mechanisms to survival patterns.

Another important factor associated with conodont survival revealed through bivariate logistic regression analyses was parameter ($\overset{-}{\gamma }$) for describing conodont environmental preferences. The standard deviations of occupied environments (*σ*(*γ*)) and the distribution skewness of occupied environments (*G*(*γ*)), which could be interpreted as rough analogues of niche breadth and shape proxy, were insignificant in predicting survival.

The multivariable logistic analyses also confirmed the importance of average gamma-ray values ($\overset{-}{\gamma }$). The regression tree analysis found this feature to be the most important determinant of extinction risk because it was selected as a criterion of the first split in the tree (Fig 9). Almost all of the studied species preferentially found in environments with very low gamma-ray values (i.e., carbonatic—shallow water environments) survived the Ireviken event. This finding suggests the existence of differential environmental stress impacts with respect to species habitat during the mass extinction event, with deeper and/or open ocean environments being disproportionately affected. This study complements previous discoveries that reveal contrasting extinction and origination dynamics between Paleozoic marine animal genera that lived in carbonate environments versus those that lived in siliciclastic environments [97]. Other studies reveal that subsequent Permian—to Mesozoic fauna from deep-ocean and shallow sea settings exhibited different forms of macroevolution kinetics [98]. Similar findings were revealed by other authors that studied planctic invertebrates from the Ordovician period (which preceded the Silurian period studied here). It was found that deep-water graptolite species exhibited higher extinction rates than those that were also found in shallow water environments [99, 100]. In addition, this environmental selectivity of conodont extinction is in agreement with the fact that taxa that suffered most during the Ireviken event (e.g., conodonts and graptolites) were more closely associated with the pelagic realm than those taxa that suffered the least (polychaets and brachiopods), which are typically referred to as truly benthic.

The open ocean pelagic realm may have been more vulnerable because it is much more homogenous than benthic substrates. Hence, during strong climatic perturbations it may easily experience a sudden loss of dynamic structuring in bathymetrically and geographically defined habitats, generating devastating consequences for pelagic species [101, 102].

The Ireviken extinction event, which is the focus of this study, as originally envisioned by Jeppsson [4, 5], resulted from self-organizing interaction between ocean-atmosphere-biota-sediments systems that was affected to some degree by Milankovitch cycles. These dynamics resulted in the intercalation of different ocean and atmosphere states of differing climates in so-called “Primo” (humid) and “Secundo” (arid) episodes. The “Primo” episodes were characterized by cold, energetic and high latitude water-driven vertical ocean convection and a generally cold and humid climate. The “Secundo” episodes were characterized by mid-latitude, saltwater-driven, sluggish oceanic convection, highly stratified oceans, and hot arid climates. During transitional periods between these episodes, as envisioned by the model, massive exchanges of greenhouse gases between the ocean and atmosphere occurred in conjunction with significant climatic changes. According to the model, these perturbations destroyed species habitats and impacted resource production processes that partly drove subsequent extinction events facilitated by orbital perturbations. As this model envisions, the Ireviken event represents one of the most severe manifestations of these transitions [5]. Based on a review of literature on this perturbation [4, 6, 8, 9], the event can be viewed as an example of “press-pulse” [103] environmental deterioration, in which long-term stress is enhanced by a series of relatively short and presumably periodic “beats,” which are recognized as extinction stage “datum points” or turnover episodes. However, while this model and similar models are widely supported [18], associated profound sea level perturbations [49], the rise of global temperatures (global warming), and the presence of generally unstable climates before the Sheinwodian glaciation [15] could have also played a role in this extinction event. Our findings which link preferential survival of shallow water taxa concurs with Jeppsson’s model, because they suggest that the main source of stress was probably not a sea level change per se, but changes in oceanographic conditions (i.e. changes in convection patterns).

## Conclusions

The bivariate logistic regression analyses revealed that important factors of conodont species survival during the mid-early Silurian Ireviken extinction event include abundance distribution skewness (*G*(*a*)), the spectral exponents of abundance time series (ν), and environmental preference patterns as reflected by average gamma radiation intensities in the sampling sites ($\overset{-}{\gamma }$). However, the multimodel comparison between multivariable regressions found only two factors to be influential, namely, average environmental preferences and species abundance distribution skewness. The regression tree analysis found, in addition to these two features, that rates of extirpation (*E*(*t*)) may also be influential, as they were surprisingly positively associated with species survival.

The presented findings point to the significant role of conodont species habitat affinities and temporal intraspecific abundance structures in shaping survival processes during the Ireviken extinction event. The greater impact on species that lived in the open ocean/pelagic realm suggests that changes in ocean dynamic structures rather than sea level regressions and transgressions per se were the major killing mechanisms. The statistical association between survival probability and fluctuation patterns, as reflected by (*G*(*a*)), suggests a possible positive role played by life-history strategies that mediated degrees of population growth nonlinearity in altered ecosystems during and shortly after the mass extinction event. Moreover, the positive relationship between species abundance distribution skewness and survival probabilities may have arisen as a consequence of theoretically expected associations between this feature and the generation of higher degrees of phenotypic variability during episodes of unchecked population growth.

## Supporting Information

S1 File Excel file contains information about abundance of conodont elements, and also gamma-ray intensities in samples of five deep cores. (XLS)

S2 File Excel file contains values of ten predictors which were used in the study of conodont extinction selectivity. (XLS)
